# Evaluating the Cancer Therapeutic Potential of Cardiac Glycosides

**DOI:** 10.1155/2014/794930

**Published:** 2014-05-08

**Authors:** José Manuel Calderón-Montaño, Estefanía Burgos-Morón, Manuel Luis Orta, Dolores Maldonado-Navas, Irene García-Domínguez, Miguel López-Lázaro

**Affiliations:** ^1^Department of Pharmacology, Faculty of Pharmacy, University of Seville, 41012 Seville, Spain; ^2^Department of Cell Biology, Faculty of Biology, University of Seville, Spain

## Abstract

Cardiac glycosides, also known as cardiotonic steroids, are a group of natural products that share a steroid-like structure with an unsaturated lactone ring and the ability to induce cardiotonic effects mediated by a selective inhibition of the Na^+^/K^+^-ATPase. Cardiac glycosides have been used for many years in the treatment of cardiac congestion and some types of cardiac arrhythmias. Recent data suggest that cardiac glycosides may also be useful in the treatment of cancer. These compounds typically inhibit cancer cell proliferation at nanomolar concentrations, and recent high-throughput screenings of drug libraries have therefore identified cardiac glycosides as potent inhibitors of cancer cell growth. Cardiac glycosides can also block tumor growth in rodent models, which further supports the idea that they have potential for cancer therapy. Evidence also suggests, however, that cardiac glycosides may not inhibit cancer cell proliferation selectively and the potent inhibition of tumor growth induced by cardiac glycosides in mice xenografted with human cancer cells is probably an experimental artifact caused by their ability to selectively kill human cells versus rodent cells. This paper reviews such evidence and discusses experimental approaches that could be used to reveal the cancer therapeutic potential of cardiac glycosides in preclinical studies.

## 1. Introduction

**Figure 1:**
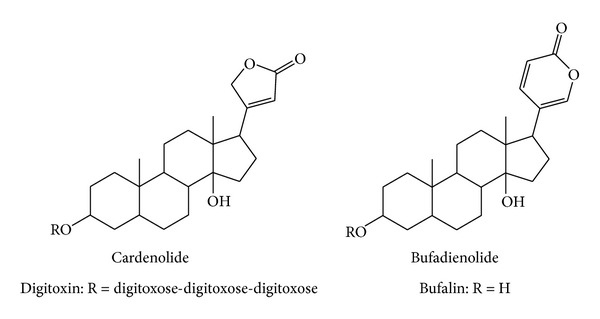
Chemical structure of cardiac glycosides. The basic skeletons of cardenolides and bufadienolides and the structures of the cardenolide digitoxin and the bufadienolide bufalin are shown.

Cardiac glycosides, also known as cardiotonic steroids, are natural products with a steroid-like structure and an unsaturated lactone ring. They usually contain sugar moieties in their structure and have cardiotonic activity. Cardiac glycosides containing the lactone 2-furanone are known as cardenolides and those containing the lactone 2-pyrone are known as bufadienolides (Figure 1). Most cardiac glycosides (e.g., digitoxin, digoxin, ouabain, and oleandrin) have been isolated from plants, including* Digitalis purpurea, Digitalis lanata*,* Strophanthus gratus*, and* Nerium oleander*. Some cardiac glycosides have also been found in amphibians and mammals, including digoxin, ouabain, bufalin, marinobufagenin, and telecinobufagin. Several cardiac glycosides are used in cardiology for the treatment of cardiac congestion and some types of cardiac arrhythmias. The mechanism by which these drugs affect cardiac contractility is thought to be mediated by a highly specific inhibition of the Na^+^/K^+^-ATPase pump [1–3].

Over the years, several reports have suggested that cardiac glycosides may have an anticancer utilization (reviewed in [4–13]).* In vitro* and* ex vivo* experiments have revealed that some cardiac glycosides (e.g., digitoxin) induce potent and selective anticancer effects [4, 14, 15], which may occur at concentrations commonly found in the plasma of patients treated with these drugs [16]. Recent high-throughput screenings of drug libraries have identified several cardiac glycosides (e.g., digoxin, ouabain, and bufalin) as potent inhibitors of cancer cell growth [17–19]. These cardiac glycosides were also able to block tumor growth in mice xenotransplanted with human cancer cells, further supporting the idea that these compounds should be evaluated in cancer patients [17–19]. The cardiac drugs digitoxin and digoxin, the semisynthetic cardiac glycoside UNBS1450, and two extracts from the plant* Nerium oleander* have entered clinical trials for the treatment of cancer (see http://clinicaltrials.gov/ and ref. [6, 7, 10, 20, 21]).

Research results also suggest, however, that cardiac glycosides may not inhibit cancer cell proliferation selectively in particular types of cancer [22–24] and the potent inhibition of tumor growth induced by cardiac glycosides in mice xenografted with human cancer cells is probably an experimental artifact caused by their ability to selectively kill human cells versus rodent cells rather than by their ability to selectively kill human cancer cells versus human normal cells [24–26]. After reviewing such evidence, this paper discusses experimental approaches that can be used to reveal the cancer therapeutic potential of cardiac glycosides in preclinical studies.

## 2. Possible Misinterpretation of Data from Preclinical Studies

Inhibition of cancer cell proliferation at low concentrations and inhibition of tumor growth in animal models are the most common parameters used by researchers to assess the therapeutic potential of drug candidates in preclinical studies. Based on this approach, researchers have proposed cardiac glycosides as candidates for evaluation in clinical trials. This section of the paper reviews evidence indicating that this approach may be inadequate to reveal the cancer therapeutic potential of cardiac glycosides.

### 2.1. Inhibition of Cancer Cell Proliferation at Low Concentrations Does Not Reliably Predict Therapeutic Potential

The key feature of an efficient anticancer drug candidate is its ability to kill (or to inhibit the proliferation of) human cancer cells at concentrations that do not significantly affect human nonmalignant cells. If the anticancer drug candidate does not have this feature, it does not really matter whether or not it can kill cancer cells at low concentrations. The reason is that the drug concentrations required to kill the tumor cells of cancer patients would also cause the death of their normal cells and, therefore, would be lethal to these patients. It is important to note that the therapeutic potential of a drug able to kill cancer cells at a concentration of 1 millimolar without significantly affecting nonmalignant cells at a concentration of 10 millimolar is probably higher than that of a drug that kills both cancer and nonmalignant cells at a concentration of 1 nanomolar.

Cancer researchers do not commonly use human nonmalignant cells to assess the therapeutic potential drug candidates. Possible reasons are that they may consider that the inhibition of human cancer cell proliferation at low concentrations is an adequate parameter to predict therapeutic potential or they prefer using animal models instead. Researchers typically use mice xenotransplanted with human cancer cells to reveal whether their drug candidates inhibit cancer cell growth selectively. If their drugs inhibit tumor growth in these models without killing or significantly affecting the animals, they assume that their drugs also inhibit the proliferation of human cancer cells without significantly affecting that of human nonmalignant cells. Following this approach, researchers have proposed several cardiac glycosides as candidates for clinical testing in cancer patients [17–19, 27, 28].

Several research groups have evaluated the cancer therapeutic potential of cardiac glycosides by using human cancer cells and human nonmalignant cells. For instance, we recently observed that the cytotoxicity of digitoxin, digoxin, and ouabain in breast cancer cells (MCF-7) and melanoma cells (UACC-62) was similar than that in nonmalignant breast cells (MCF-10) and nonmalignant skin cells (VH-10) [24]. Clifford and Kaplan [23] have recently reported that human breast cancer cells were even more resistant to ouabain, digitoxin, and bufalin toxicity than human nonmalignant breast cells. Evidence has also shown, however, that digitoxin, digoxin, and ouabain were approximately 10 times more cytotoxic against human A549 lung cancer cells than against human MRC-5 nonmalignant lung cells (5–8 nM versus 29–75 nM) [24].* Ex vivo* experiments, using cells from adult patients with B-precursor or T-acute lymphoblastic leukemia (ALL), acute myeloid leukemia (AML), and chronic lymphocytic leukemia (CLL), as well as peripheral blood mononuclear cells from healthy donors, have also shown that digitoxin (but not ouabain) induced selective cytotoxicity (approximately 7-fold) in cells from patients with T- and B-precursor ALL [15]. In brief, although cardiac glycosides can inhibit the proliferation of cancer cells at very low concentrations (nM), they usually inhibit the proliferation of human nonmalignant cells at similar concentrations; this strongly suggests that their potential for cancer therapy is low. In contrast, specific cardiac glycosides (e.g., digitoxin) can inhibit the proliferation of particular types of cancer cells (e.g., lung cancer and acute lymphoblastic leukemia) at concentrations that do not significantly affect human nonmalignant cells; these cardiac glycosides may have cancer therapeutic potential.

### 2.2. The Anticancer Activity of Cardiac Glycosides in Mice Xenografted with Human Cancer Cells Is Probably an Experimental Artifact

Several cardiac glycosides that are equally toxic to human cancer cells and human nonmalignant cells have shown potent anticancer effects in animal models. For instance, Clifford and Kaplan observed that human nonmalignant breast cells were more sensitive than human breast cancer cells (e.g., MDA-MB-231) to the cytotoxic effects of bufalin [23], and it has recently been reported that bufalin reduces tumor growth in mice xenotransplanted with human MDA-MB-231 breast cancer cells [19]. These apparent controversies can be explained by the ability of cardiac glycosides to kill human cells at concentrations much lower (approximately 100–1000 fold) than those required to kill rodent cells [24, 29].

Gupta and colleagues [29] evaluated some time ago the cytotoxicity of numerous cardiac glycosides (i.e., ouabain, digitoxin, digoxin, convallatoxin, SC4453, bufalin, gitaloxin, digoxigenin, actodigin, oleandrin, digitoxigenin, gitoxin, strophanthidin, gitoxigenin, lanatosides A, B, and C, alpha- and beta-acetyl digoxin, and alpha- and beta-methyl digoxin) against a number of independent cell lines established from human, monkey, mouse, Syrian hamster, and Chinese hamster. The authors observed that all cardiac glycosides exhibited greater than 100-fold higher toxicity towards the human and monkey cells in comparison to the rodent cells (mouse, Syrian hamster, and Chinese hamster). They also provided strong evidence that the species-related differences in sensitivity to cardiac glycosides were mediated by the Na^+^/K^+^-ATPase enzyme. They observed that the Na^+^/K^+^-ATPase enzyme of rodent cells was inhibited at much higher concentrations of cardiac glycosides than the Na^+^/K^+^-ATPase of human cells. They also observed a good correlation between these concentrations and those reported for inhibition of the Na^+^/K^+^-ATPase from isolated heart muscles of the same species [29]. More recent evidence suggests that the expression and cellular location of Na^+^/K^+^-ATPase alpha subunits in different types of cells may explain why they are more or less susceptible to the cytotoxic activity of cardiac glycosides [30–32].

Several years ago, a PNAS paper reported that digoxin blocked tumor growth in mice xenotransplanted with several types of human cancer cells [17]. The authors observed that digoxin prolonged tumor latency and inhibited tumor xenograft growth in mice when treatment was initiated before the implantation of P493-Myc, P493-Myc-Luc, PC3, and Hep3B cells. Digoxin also arrested tumor growth when treatment was initiated after the establishment of PC3 and P493-Myc tumor xenografts [17]. Based on the observations of Gupta and colleagues [29] and on the plasma levels of digoxin in cardiac patients, we discussed the fact that the potent anticancer effects induced by digoxin in mice harboring human cancer cells [17] were not relevant to the treatment of human cancer and these anticancer effects were probably due to interspecies differences in sensitivity [25]. In other words, the marked reduction in tumor growth induced by digoxin in mice xenografted with human cancer cells was probably caused by the ability of cardiac glycosides to selectively kill human cells versus rodent cells rather than by their ability to selectively kill cancer cells versus normal cells. Perne et al. [22] later reported experimental data that further supported this idea. Despite these and other reports [24, 26], numerous publications containing this probable experimental artifact continue to appear in the scientific literature.

This section of the paper now reviews reports that have used mice xenotransplanted with human cancer cells to evaluate anticancer effects of cardiac glycosides (Table 1). The results of the following reports should probably be reinterpreted.

*Digoxin.* Svensson et al. [33] carried out* in vitro *and* in vivo *studies to evaluate the anticancer activity of the cardenolide digoxin. They studied the effect of digoxin on the growth of tumor cell lines and primary endothelial cells from different species. The most sensitive cell lines* in vitro* were the human SH-SY5Y and SK-N-AS neuroblastoma cell lines; the IC_50_ values were 34 and 22 ng/mL, respectively. They also reported that digoxin significantly reduced the growth of human SH-SY5Y neuroblastoma cells xenotransplanted in immunodeficient mice. The authors concluded that digoxin might be a specific neuroblastoma growth inhibitor. The authors also reported that the* in vitro* and* in vivo* anticancer effects of digoxin were dramatically reduced when the murine Neuro-2a neuroblastoma cell line was used instead of the human neuroblastoma cell lines [33]. Zavareh et al. [34] reported data suggesting that cardiac glycosides were inhibitors of N-glycan biosynthesis. Since aberrant N-linked glycans are known to contribute to cancer progression and metastasis, the authors studied whether digoxin could inhibit cellular migration and invasion. They used two mouse models of metastatic cancer in which human PPC-1 prostate cancer cells were injected into immunodeficient mice. They found that digoxin reduced distant tumor formation in both models and concluded that this cardiac glycoside could be a lead for the development of antimetastasis therapies. As discussed before, Zhang et al. [17] found that digoxin inhibited hypoxia-inducible factor 1 (HIF-1), a transcription factor highly involved in cancer development, and suggested that this effect might be observed in patients taking this drug. They also reported that digoxin blocked tumor growth in mice xenotransplanted with several types of human cancer cells. These data suggested that digoxin had anticancer potential [17]. Wong et al. [35] reported data suggesting that digoxin was a potential antimetastasis compound. They investigated whether digoxin could reduce metastases in human MDA-MB-435 tumor-bearing mice. Digoxin blocked metastatic niche formation and breast cancer metastasis in the lungs, and the authors discussed the fact that this effect was probably due to inhibition of HIF-1. The most relevant conclusion of this work was that digoxin might be useful to treat patients with HIF-1-overexpressing breast cancers [35]. Zhang et al. [36] observed that digoxin reduced tumor growth and inhibited the metastasis of human MDA-MB-231 breast cancer cells to the lungs in mice xenografted with these cells, without causing any sign of toxicity in the animals. They concluded that clinical trials were warranted to investigate whether the concentrations of digoxin achievable in patients are sufficient to inhibit tumor growth and metastases [36]. Schito et al. [37] reported that HIF-1 promoted lymphatic metastases of breast cancer and the use of the HIF-1 inhibitor digoxin strongly decreased tumor growth and blocked lymphangiogenesis and lymphatic metastasis in mice bearing human breast cancer cells. The authors suggested that digoxin might be useful to treat patients with high risk of lymphatic metastases [37]. Gayed et al. [38] observed that specific concentrations of digoxin inhibited blood vessel formation but not tumor growth in mice injected with the human C4-2 prostate cancer cell line.

*Ouabain.* Several cardiac glycosides were identified by Antczak et al. [39] as potent antiretinoblastoma agents* in vitro*. One of them, the cardenolide ouabain, induced a drastic tumor regression in immunodeficient mice injected with human Y79LUC retinoblastoma cells, without inducing any significant toxicity on the host. In light of the results of their study, the authors proposed that digoxin, which is widely used in patients with cardiac disease, could be repositioned for the treatment of retinoblastoma [39]. Simpson et al. [40] identified the cardiac glycosides ouabain, peruvoside, digoxin, digitoxin, and strophanthidin as anoikis sensitizers. Because resistance to anoikis permits cancer cells to survive in the circulation and improves their metastatic potential, the authors evaluated in mouse models of metastasis whether ouabain could block distant tumor formation. They observed that ouabain reduced the number of tumors in human PPC-1 prostate cancer cells bearing mice. They also reported that systemic administration of ouabain decreased the survival and growth of human PPC-1 prostate cancer cells and human BON1 pancreatic cancer cells xenografted into nude mice [40]. Hiyoshi et al. [27] reported that ouabain induced quiescence in neuroblastoma cells* in vitro* and a marked reduction in tumor growth when human neuroblastoma cells were xenografted into immune-deficient mice. Based on these findings, the authors concluded that ouabain could be used in chemotherapies to suppress tumor growth and/or arrest cells to increase the therapeutic index in combination therapies. Tailler et al. [18] identified the cardiac glycoside ouabain as a potential antileukemic compound. They observed that ouabain was highly efficient in inhibiting the growth of human acute myeloid leukemia cells xenotransplanted in immunodeficient mice, without exerting significant toxicity on the host. The authors concluded that ouabain was a promising antileukemic agent whose activity should be evaluated in prospective clinical studies [18].

*UNBS1450.* Mijatovic et al. [41] investigated the* in vitro* and* in vivo* anticancer activity of UNBS1450, a semisynthetic derivative of the natural cardenolide UNBS1244 (isolated from the African plant* Calotropis procera*). They observed that UNBS1450 was able to inhibit cell growth of four different non-small cell lung cancer cells (A549, NCI-H727, A427, and CAL-12T) at nanomolar concentrations. This cardenolide also significantly decreased tumor growth in nude mice xenografted with human NCI-H727 cancer cells and increased the survival rates in mice xenografted with human A549 cancer cells. The authors observed in another study [42] that the cytotoxic potency of UNBS1450 in A549 lung cancer cells was similar than that of the anticancer drugs paclitaxel and SN38 (the active metabolite of irinotecan) and much higher than that of cisplatin, carboplatin, and oxaliplatin. UNBS1450 also decreased tumor growth in mice xenotransplanted with A549 lung cancer cells and human NCI-H727 lung cancer cells [42]. Another study revealed that UNBS1450 inhibited the proliferation of human prostate cancer cells (LNCaP, PC-3, and DU145) and increased the survival of mice transplanted with human PC-3 prostate cancer cells [43]. Lefranc et al. [44] reported that UNBS1450 was more cytotoxic on human glioblastoma cells (U373-MG and T98G) than on human normal fibroblasts (WI-38 and WSI) at nanomolar concentrations. This compound also inhibited the proliferation of rat C6 glioblastoma cells at micromolar concentrations. UNBS1450 increased the survival of mice grafted with human U373-MG glioblastomas cells, without observable toxic effects on the animals. Mathieu et al. [45] reported that UNBS1450 blocked cell proliferation in several human melanoma cell lines* in vitro* (IC_50_ values between 5 and 45 nM) and improved the survival of immunodeficient mice grafted with human VM-48 melanoma brain metastasis cells.

*Periplocin.* Lu et al. [46] reported that the natural cardenolide periplocin induced similar cytotoxicity against a panel of human lung cancer cell lines than against a rodent lung cancer cell line (LL/2). They also observed antitumor activity in mice transplanted with both the human A549 lung cancer cell line and the murine LL/2 Lewis lung cancer cell line. Cheng et al. [47] have recently reported that periplocin displayed a potent cancer cell growth inhibitory activity* in vitro* and* in vivo*. Periplocin inhibited cell growth of human HA22T/VGH hepatocellular carcinoma with an IC_50_ of 27 nM and was less toxic to normal peripheral blood mononucleated cells. The authors also observed that periplocin showed an inhibition of tumor growth when human Huh-7 hepatoma cells were injected into immunodeficient mice, without observing clear side effects on the host.

*Lanatoside C.* Badr et al. [48] identified the cardenolide lanatoside C as a sensitizer of glioblastoma cells to tumor necrosis factor-related apoptosis-inducing ligand (TRAIL)-induced cell death. They observed that lanatoside C, alone or in combination with TRAIL, reduced tumor growth in nude mice harboring human U87 glioblastoma cells.

*Bufalin.* Chen et al. [49] identified the bufadienolide bufalin as a potential agent for the treatment of pancreatic cancer in combination with the standard anticancer drug gemcitabine. They found that bufalin inhibited the growth on three pancreatic cancer cell lines (Bxpc-3, Mia PaCa-2, and Panc-1) and it synergistically increased gemcitabine-induced cancer cell growth inhibition and apoptosis. The combination of bufalin with gemcitabine was also found to significantly reduce tumor growth in mice bearing human Mia Paca-2 pancreatic cancer cells. Xie et al. [50] investigated the* in vitro* and* in vivo *antiosteosarcoma activity of bufalin. They observed that bufalin strongly inhibited the cell growth of different human osteosarcoma cell lines, including the methotrexate-resistant U2OS/MTX300 cell line. They also found that the treatment with bufalin induced significant tumor growth inhibition in mice xenotransplanted with the human U2OS/MTX300 osteosarcoma cell line, without decreasing the body weight of the animals. The authors concluded that bufalin might be an alternative chemotherapeutic agent to treat osteosarcoma, particularly in methotrexate-resistant cancers [50]. Wang et al. [19] have recently reported that bufalin was a potent inhibitor of the steroid receptor coactivators SRC-3 and SRC-1. Because these coactivators have been implicated in cancer progression, the authors investigated whether bufalin could also block cancer cell growth in cell culture and animal models. They observed that bufalin inhibited the growth of human MCF-7 breast cancer cells and human A549 lung cancer cells at nanomolar concentrations (3–5 nM); these concentrations also resulted in inhibition of the steroid receptor coactivator SRC-3 and were below those reported to be tolerated by humans (8.75 nM). They also found that bufalin inhibited tumor growth in mice xenotransplanted with human MDA-MB-231 breast cancer cells.

*Arenobufagin.* Zhang et al. [28] recently observed that the bufadienolide arenobufagin induced a potent cell growth inhibitory activity on cancer cells both* in vitro *and* in vivo*. They tested its anticancer activity on several human cancer cell lines (hepatoma, breast adenocarcinoma, cervix adenocarcinoma, lung cancer, colon cancer, leukemia, and gastric adenocarcinoma). Arenobufagin inhibited the growth of all cancer cell lines at nanomolar concentrations, including multidrug-resistant cancer cell lines. Arenobufagin also inhibited the growth of human HepG2/ADM hepatocellular carcinoma cells xenografted into immunodeficient mice, without causing side effects on the hosts. The authors concluded that their results may provide a rationale for future clinical application using arenobufagin as a chemotherapeutic agent for the treatment of patients with hepatocarcinoma [28].

*Bufotalin.* Zhang et al. [51] observed that four bufadienolides from Venenum Bufonis, a traditional Chinese medicine, displayed inhibitory effects on the growth of human HepG2 hepatocarcinoma cells and human R-HepG2 multidrug hepatocarcinoma cells. One of them, bufotalin, was also able to significantly inhibit the growth of human R-HepG2 cells xenografted into immunodeficient mice, without observing any life-threatening toxicity in the animals. The authors discussed the fact that their study supports the possible development of bufotalin as a potential agent in the treatment of multidrug resistant hepatocellular carcinoma [51].

Data from preclinical studies reporting antitumor effects in rodent xenografts of plant extracts containing cardiac glycosides may also need reinterpretation. For instance, Han et al. [52] reported that an extract from the plant* Streptocaulon juventas* induced a strong inhibitory effect on the proliferation of human lung A549 adenocarcinoma cells. A bioassay-guided fractionation revealed that the most cytotoxic fraction* in vitro* also induced antitumor effects in athymic nude mice transplanted with human A549 cancer cells without exerting side effects on the mice. Following HPLC and NMR spectrometry, the main components of this active fraction were identified as the cardiac glycosides digitoxigenin, periplogenin, and periplogenin glucoside [52].

## 3. Possible Approaches to Reveal the Cancer Therapeutic Potential of Cardiac Glycosides in Preclinical Studies

As discussed before, the key feature of an efficient anticancer drug candidate is its ability to kill (or to inhibit the proliferation of) human cancer cells at concentrations that do not significantly affect human nonmalignant cells. Ideally, the drug candidate should kill all the cancer cells of the patients without significantly affecting their normal cells. Because this is difficult to achieve, one can settle for less. A drug that improves the ability of our current anticancer drugs to kill cancer cells at concentrations that do not significantly affect nonmalignant cells could be therapeutically useful.

*In vitro*, one can evaluate whether the drug candidate improves the selective cytotoxicity of the standard anticancer drugs towards cancer cells by using the following approach. The first step in this approach is the selection of a panel of human cancer cell lines and human nonmalignant cell lines (or primary cells). Because the cytotoxicity of some drugs depends on the nature of the tissue from which they originate, one should select nonmalignant cell lines of the same tissue origin than that of the selected cancer cell lines. A small number of cancer cell lines may be sufficient to reveal the therapeutic potential of a drug for a particular type of cancer. However, the selection of a low number of nonmalignant cell lines reduces the chances of finding toxicity on a specific tissue that would limit the possible therapeutic use of the drug. The next step is to treat the selected cell lines with several concentrations of the drug candidate and of the anticancer drugs most commonly used in the treatment of the selected cancers. Then, cell viability or cell death is estimated with a cytotoxicity test (e.g., SRB assay and MTT assay), and cytotoxic parameters (e.g., IC_50_ values) are calculated. The following step is to calculate one or several selectivity indexes for the drug candidate and for the anticancer drug. These selectivity indexes can be calculated by dividing the IC_50_ values in the nonmalignant cell lines by the IC_50 _values in the cancer cell lines. For instance, if the mean IC_50_ value of a drug in a variety of nonmalignant cells originated from several tissues is 100 *μ*M and the mean IC_50_ value of the drug in several cell lines derived from a specific cancer is 20 *μ*M, the selectivity index for this particular cancer would be 5. Finally, the following question must be answered: is the selectivity index of the drug candidate higher (or at least similar) than that of the standard anticancer drug? If the answer is no, the drug candidate does not have therapeutic potential and should not be tested in animal models. If the answer is yes, the drug candidate has chemotherapeutic potential, which should be confirmed by using* in vivo* experiments.

Rodent xenograft models are the most common animal models used by researchers to evaluate the therapeutic potential of anticancer drug candidates* in vivo*. However, as discussed before, these models may be inadequate to evaluate the therapeutic potential of cardiac glycosides. To the authors' knowledge, all cardiac glycosides tested in human cells and rodent nonmalignant cells have shown greater than 100-fold higher toxicity towards the human cells in comparison to the rodent cells. This does not mean, however, that all compounds having the basic chemical structure of cardiac glycosides (a steroid skeleton with an unsaturated lactone ring) will be more toxic against human cells than against rodent cells. One can test the suitability of using tumor xenografts to evaluate the* in vivo* therapeutic potential of a particular cardiac glycoside by testing if the cytotoxicity of the cardiac glycoside against a panel of human nonmalignant cells is similar than that against a panel of rodent nonmalignant cells. If the compound behaves similarly in both types of cell lines, its* in vivo* anticancer activity can be evaluated in mice xenografted with human cancer cells. If the rodent cell lines are more resistant than the human cell lines to the cytotoxicity of the cardiac glycoside, these models are probably inadequate to evaluate its anticancer effects* in vivo*. Animal models using mice transplanted with mouse cancer cells may also be inadequate when human cells are more sensitive than rodent cells to the cytotoxicity of the cardiac glycoside. The reason is that the therapeutic target responsible for the death of the human cells may be different than that responsible for the death of the rodent cells and, therefore, results obtained in mice may not be extrapolated to humans.

The anticancer activity of cardiac glycosides displaying a similar cytotoxic profile in nonmalignant cells originated from both human and mouse tissues can be assessed by using tumor xenografts or other rodent models. It is important to remember that most cancer patients requiring therapy with anticancer drugs have metastatic disease and patient survival is the parameter used by oncologists as an endpoint of clinical interventions designed to assess drug efficacy in patients with cancer (other parameters used by many preclinical researchers as an endpoint for their experiments, such as measurements of tumor volumes, do not necessarily predict survival). It is essential, therefore, to select animal models of metastasis and to assess animal survival as an endpoint for the experiments. In our opinion, animals with metastasis should be treated with equitoxic concentrations of the cardiac glycoside and of the standard anticancer drug used in the type of cancer under study. Then, one should evaluate whether the cardiac glycoside improves the survival rates induced by the standard anticancer drug. If the cardiac glycoside improves (or at least matches) the selectivity index (*in vitro*) and the survival rates (*in vivo*) of the standard anticancer drugs, it should be considered for clinical trials testing.

Rodent models are inappropriate for testing the anticancer activity of cardiac glycosides that kill human nonmalignant cells at lower concentrations than those required to kill rodent nonmalignant cells. These models, however, could provide information on the pharmacokinetics of the cardiac glycoside. These models may also help detect possible toxicity not detected by using a panel of human nonmalignant cell lines; they could help detect toxicity not mediated by inhibition of the Na^+^/K^+^-ATPase (which seems to be the main determinant for the species differences in sensitivity to cardiac glycosides). In our opinion, a cardiac glycoside that kills human nonmalignant cells at lower concentrations than rodent nonmalignant cells should pass the following tests before being considered for evaluation in clinical trials. First, it should match or improve the selectivity indexes of the standard anticancer drugs when they are evaluated in a panel of human cancer cell lines derived from a particular type of cancer versus a variety of human nonmalignant cell lines and primary cells derived from a variety of human tissues. Second,* in vivo* experiments (e.g., rodent models) should exclude pharmacokinetic and toxicological limitations that may compromise the* in vivo* anticancer activity of the cardiac glycoside. Finally, if the cardiac glycoside is in clinical use for the management of other diseases or if clinical data already exist on its plasma and tissue concentrations, one should also consider whether the anticancer effects observed in preclinical studies may occur at concentrations within or below the concentration range tolerated by humans.

## 4. Conclusion

Preclinical research has shown that cardiac glycosides can both inhibit cancer cell proliferation at very low concentrations and induce potent anticancer effects in mice transplanted with human cancer cells. Based on these observations, cardiac glycosides have been considered as potential anticancer drug candidates that should be evaluated in clinical studies. This paper has reviewed evidence indicating that cardiac glycosides may not selectively inhibit the proliferation of human cancer cells and these compounds have the ability of killing human cells at concentrations much lower than those required to kill rodent cells (approximately 100–1000 fold). This strongly suggests that the potent anticancer effects induced by cardiac glycosides in mice transplanted with human cancer cells may be an experimental artifact caused by their ability to selectively kill human cells versus rodent cells rather than by their ability to kill human cancer cells versus human nonmalignant cells. It has also been discussed that inhibition of cancer cell proliferation at low concentrations is not an adequate parameter to predict the therapeutic potential of a drug candidate. The key feature of an efficient anticancer drug is its ability to kill (or inhibit the proliferation of) human cancer cells at concentrations that do not significantly affect human nonmalignant cells. Based on this principle, an approach to evaluate the therapeutic potential of cardiac glycosides in preclinical* in vitro* studies has been proposed. This approach is also based on the idea that only drug candidates that match or improve the ability of the approved anticancer drugs to kill human cancer cells at concentrations that do not significantly affect human nonmalignant cells have a chance to be ultimately used in cancer therapy. A test for revealing the suitability of using rodent models for the evaluation of the anticancer activities of cardiac glycosides* in vivo* has also been proposed. If the cardiac glycoside passes this test, several recommendations have been made for the evaluation of its cancer therapeutic potential in these models. If the cardiac glycoside fails to pass this test, an alternative approach for revealing its possible therapeutic potential has been discussed. It is the hope of the authors that this paper may help researchers evaluate the therapeutic potential of cardiac glycosides in preclinical studies.

## Figures and Tables

**Table 1:** The antitumor activity of cardiac glycosides in mice xenografted with human cancer cells is probably caused by their ability to selectively kill human cells versus rodent cells rather than by their ability to selectively kill human cancer cells versus human nonmalignant cells.

Cardiac glycoside	Antitumor activity in mice xenografted with human cancer cells	Selective cytotoxicity against human cells versus rodent cells	Selective cytotoxicity against human cancer cells versus human nonmalignant cells
Arenobufagin	Liver HepG2/ADM [28]	N.D.	N.D.

Bufalin	Breast MDA-MB-231 [19], osteosarcoma U2OS/MTX300 [50], and pancreatic Mia Paca-2 [49]	>1000-fold [29]	NO: breast cancer versus breast nonmalignant [23]; 10-fold: ovarian cancer versus endometrial nonmalignant [53]

Bufotalin	Liver R-HepG2 [51]	>100-fold [54]	N.D.

Digitoxin	N.D.	>1000-fold [29]; >700-fold [24]	10-fold: lung cancer versus lung nonmalignant [24]; 3-fold: lung cancer versus lung nonmalignant [55]; 7-fold: ALL versus PBMCs nonmalignant [15]; 4-fold: AML versus PBMCs nonmalignant [15]; 2-fold: CLL versus PBMCs nonmalignant [15]; 2-fold: breast cancer versus breast nonmalignant [24]; NO: breast cancer versus breast nonmalignant [23]; NO: skin cancer versus skin nonmalignant [24]

Digoxin	Brain SH-SY5Y [33], brain SK-N-AS [33], breast MDA-MB-231 [36, 37], breast MDA-MB-435 [35], liver Hep3B [17], prostate PC3 [17], prostate PPC-1 [34], and transformed human B-lymphocytes P493-Myc [17]	>1000-fold [24, 29]	NO: breast cancer versus breast nonmalignant [24]; 8- fold: lung cancer versus lung nonmalignant [24]; NO: skin cancer versus skin nonmalignant [24]; 8-fold: brain cancer versus umbilical vein endothelial nonmalignant [33]; NO: breast cancer versus umbilical vein endothelial nonmalignant [33]; NO: colorectal cancer versus umbilical vein endothelial nonmalignant [33]

Lanatoside C	Brain U87 [48]	>100-fold [29]	N.D.

Ouabain	Brain SH-SY5Y [27], ocular Y79LUC [39], pancreatic BON1 [40], promyelocytic leukemia HL-60 [18], and prostate PPC-1 [40]	>1000-fold [24, 29, 44]	NO: breast cancer versus breast nonmalignant [23, 24]; 5- fold: lung cancer versus lung nonmalignant [24]; NO: skin cancer versus skin nonmalignant [24]; 2-fold: ALL versus PBMCs nonmalignant [15]; 2-fold: AML versus PBMCs nonmalignant [15]; NO: CLL versus PBMCs nonmalignant [15]

Periplocin	Liver Huh-7 [47] and lung A549 [46]	>1000-fold [47]*; NO: [46]	>1000-fold: liver cancer versus PBMCs nonmalignant [47]*

UNBS1450	Brain U373-MG [44], lung A549 [41, 42], lung NCI-H727 [41, 42], prostate PC-3 [43], and skin VM-48 [45]	>100-fold [44]	10-fold: brain cancer versus lung and skin nonmalignant [44]; 100-fold: prostate cancer versus lung and skin nonmalignant [43]

N.D.: not determined; NO: no selective cytotoxicity; ALL: acute lymphoblastic leukemia; AML: acute myeloid leukemia; CLL: chronic lymphocytic leukemia; PBMCs: peripheral blood mononuclear cells; *not specified if the PBMCs were human cells or rodent cells (we contacted the authors without success).
